# A minimum functional form of the *Escherichia coli* BAM complex constituted by BamADE assembles outer membrane proteins *in vitro*

**DOI:** 10.1016/j.jbc.2024.107324

**Published:** 2024-04-25

**Authors:** Zhe Wang, Yindi Chu, Qingrong Li, Xiaochen Han, Leyi Zhao, Hanqing Zhang, Kun Cai, Xuyan Zhang, Xingyuan Wang, Youcai Qin, Enguo Fan

**Affiliations:** 1 Department of Microbiology and Parasitology, Institute of Basic Medical Sciences Chinese Academy of Medical Sciences, School of Basic Medicine Peking Union Medical College, Beijing, China; 2 School of Medicine, Linyi University, Linyi, China

**Keywords:** β-barrel assembly machinery (BAM), outer membrane proteins, *in vitro* reconstitution, protein integration, membrane protein biogenesis

## Abstract

The biogenesis of outer membrane proteins is mediated by the β-barrel assembly machinery (BAM), which is a heteropentomeric complex composed of five proteins named BamA-E in *Escherichia coli*. Despite great progress in the BAM structural analysis, the molecular details of BAM-mediated processes as well as the exact function of each BAM component during OMP assembly are still not fully understood. To enable a distinguishment of the function of each BAM component, it is the aim of the present work to examine and identify the effective minimum form of the *E. coli* BAM complex by use of a well-defined reconstitution strategy based on a previously developed versatile assay. Our data demonstrate that BamADE is the core BAM component and constitutes a minimum functional form for OMP assembly in *E. coli*, which can be stimulated by BamB and BamC. While BamB and BamC have a redundant function based on the minimum form, both together seem to cooperate with each other to substitute for the function of the missing BamD or BamE. Moreover, the BamA^E470K^ mutant also requires the function of BamD and BamE to assemble OMPs *in vitro*, which *vice verse* suggests that BamADE are the effective minimum functional form of the *E. coli* BAM complex.

Outer membrane proteins (OMPs) that are embedded within the outer membrane (OM) of Gram-negative bacteria are unique because of their β-barrel structures, which are formed by even numbers of β-strands that fold into a cylinder by hydrogen bonds between the first and the last one (1, 2). These OMPs are synthesized in cytoplasmic ribosomes and have to be transported across the inner membrane by the Sec-translocon before being integrated into the OM (3). With the help of periplasmic chaperones like SurA or Skp/DegP, OMPs maintain an OM-integration-competent state at the periplasmic space and are subsequently assembled correctly into the OM by the β-barrel assembly machinery (BAM) (4), which is highly conserved in function but variable in compositions in different bacteria (5, 6).

In γ-proteobacteria like *Escherichia coli* (*E. coli*), the BAM complex is composed of five proteins: BamA, BamB, BamC, BamD, and BamE (7). While in β-proteobacteria like *Neisseria meningitides*, the BAM complex contains BamA, BamC, BamD, and BamE, which differs from that of *E. coli* by the absence of BamB homolog (8). Similarly, in all sequenced α-proteobacteria, BamC or a homologous protein is missing (5). Fewer components are found in the pathogen of Lyme disease-*Borrelia burgdorferi*, whose BAM complex consists of only three components: BB0795 (BamA), BB0028 (BamB), and BB0324 (BamD) (9). Furthermore, in *Thermus thermophilus*, which is considered an evolutional intermediate between Gram-positive and Gram-negative bacteria, only one BAM component, *Tt*Omp85 was identified (10, 11). Moreover, the continuous evolution leads to the formation of three subunits constituted BAM homologs in the OM of eukaryotic organelles: the SAM complex (Sam50, Sam35, and Sam37) in mitochondria (12, 13, 14) and the TOC complex (Toc75, Toc34, and Toc159) in chloroplasts (15). These discrepancies in the BAM composition suggest that auxiliary *E. coli* BAM lipoproteins are likely disposable or redundant in the assembly process of OMPs except BamA, which is indispensable and highly conserved in evolution since its homologs have been found existing in the OM of all Gram-negative bacteria as well as the mitochondria and chloroplasts of eukaryotes (12, 16, 17).

BamA contains a β-barrel structure that comprises 16 β-strands, and an N-terminal periplasmic region called polypeptide transport-associated (POTRA) domain with a number of variations (from 1-7) in different species (18, 19). BamA is the essential and only component within the BAM complex that traverses the OM with its β-barrel domain forming a lateral gate to enable the folding and insertion of incoming OMPs (20, 21, 22, 23). Among the other four lipoproteins in *E. coli*, BamD is the most conservative one (24, 25, 26, 27) and genetic deletion experiments have shown that BamD is crucial for bacterial cell survival (24, 28). Its N- and C-terminal regions are in contact with the POTRA 2 and POTRA 5 of BamA, respectively (29), and are likely to mediate the recognition of unfolded OMPs or to activate BamA, two hypotheses both require further extensive investigations (30, 31). Furthermore, BamD also directly interacts with BamE, a non-essential BAM component for cell viability, to form an interface between BamA/BamD (32), *via* which it is believed that BamE functions as a regulator of BamD (33). Moreover, recent observation suggests that BamE also interacts directly with BamA and has a coordinated function for the activities of BamA and BamD (34). While BamB and BamC are not essential components of the BAM complex as their single gene knockout in *E. coli* strain does not affect cell growth (35), the function of BamB is believed to serve as a regulator for BamA where the interaction occurs at the hinge region between POTRA 2 and POTRA 3 of BamA (36). BamC is the most mysterious component of the BAM complex and its function alone remains elusive. A cartoon illustrating the interactions among *E. coli* BAM components is shown in Figure 1*A*.

**Figure 1:**
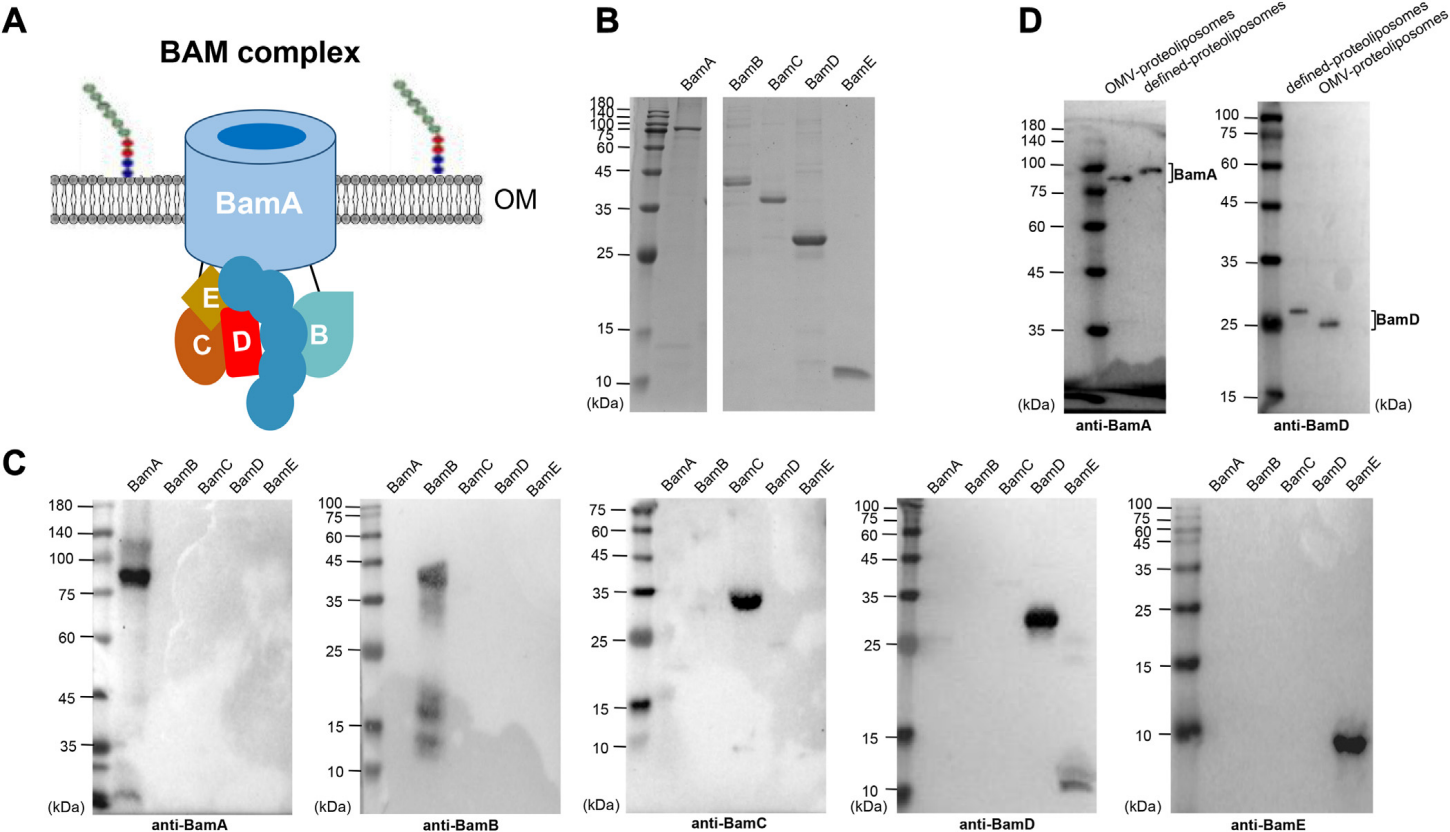
**The *E. coli* BAM complex.***A*, cartoon of the composition of the *E. coli* BAM complex and the relative interaction of each component were according to the Ref. (65). *B*, 7.2 × 10^−5^ μmol BamA-E proteins were loaded on a 15% SDS-PAGE gel and stained with Coomassie Blue R-250. The molecular weights of purified recombinant BamA, B, C, D, and E proteins are nearly consistent with the theoretical sizes (BamA 94.1 kDa, BamB 45.4 kDa, BamC 40.4 kDa, BamD 31.3 kDa, and BamE 15.8 kDa). *C*, cross-reaction detection among BamA-E proteins by western-blot analysis using anti-BamA, BamB, BamC, BamD, and BamE antibodies. *D*, quantification of defined-proteoliposomes and OMV-proteoliposomes by using BamA and BamD as the reference proteins. It was performed by immunoblotting using anti-BamA and BamD antibodies.

Despite the most extensive investigations on BamA that form the basis of our current understanding of the molecular mechanism of OMP assembly, the exact functions of the other individual BamB-E components during OMP assembly require further investigations. Moreover, a stable BAM holo-complex seems to be not required for its assembly function because a separation into BamAB and BamDCE subcomplexes is still functional when genetic mutations are placed at the BamA/BamD interface (37, 38). Most interestingly, further mutations at BamA (*i.e.*, BamA^E470K^) seem to relieve the requirement of the BAM lipoproteins *in vivo* (33, 39, 40), therefore indicating that this BamA mutant might be able to assemble OMPs in the absence of other BAM lipoproteins. Interestingly, naturally, the BamA homolog *Tt*Omp85 alone has been proven to function as a translocase and an insertase that is capable of substituting the function of the five subunits comprised of *E. coli* BAM complex (41). Altogether, these data suggest that the lipoproteins BamB-E likely play only regulatory roles in *E. coli*, that is, as hypothesized that BamB regulates BamA and BamE/BamC regulates BamD (33) or some other yet-to-be-identified functions. Therefore, to understand the exact function and the necessity of each BAM component in facilitating the assembly of OMPs in *E. coli*, it is essential to first figure out the effective minimum functional form of the BAM complex and then analyze the potential redundant functions among various BAM components. Based on our previously developed versatile *in vitro* reconstitution system (42, 43), in the present work, we used various combinations of extensively purified *E. coli* BamA-E proteins to examine the effective minimum functional form of the BAM complex and then to clarify the redundancy of the BAM components. Our results demonstrate that although no single/double combinations of the BAM components showed any assembly function, three-component combinations of BamADE gave rise to a successful effective assembly of the model substrate OmpA (containing 8 β-strands). Surprisingly, the BamA^E470K^ mutant alone cannot assemble OmpA *in vitro* and still requires BamD+BamE to function. Furthermore, large OMPs, that is, BamA protein (containing 16 β-strands) itself can also be assembled by the minimum functional form comprised by BamADE, suggesting that this minimum form is not limited to small OMPs despite four-component-combinations enabling a better assembly of large OMPs. Further examinations suggest that both BamB and BamC increased the assembly efficiency in the assembly of OmpA and BamA and showed a redundant function. Our results also suggest that BamB and BamC are likely able to cooperate with each other to substitute for the function of the missing component of BamD or BamE. Thus, our results provide an overview of the redundancy and potential functional combinations of the *E. coli* BAM components during the assembly process of OMPs.

## Results

### Purification and validation of each *E. coli* BAM component

To enable a clean outer membrane environment to examine how each BAM component contributes to the assembly of OMPs, we sought to use extensively purified BAM proteins to construct well-defined proteoliposomes based on our versatile system developed previously (42). The purified BamA-E proteins (7.2 × 10^-5^ μmol) were prepared and loaded on a 15% SDS-PAGE gel to check their purities, and as shown in Figure 1*B* all five proteins run at their predicted theoretical molecular weights. To confirm the identity of the purified BAM proteins and to examine their purities further, a specific antibody against each BAM component was used to perform western-blot analysis. As shown in Figure 1*C*, it is clear that no cross-reaction among BamA-E proteins was observed, demonstrating that there is no observed mutual contamination among the purified proteins, and thus any observed effects in subsequent experiments should be attributed to any combined BAM components.

**Figure 2:**
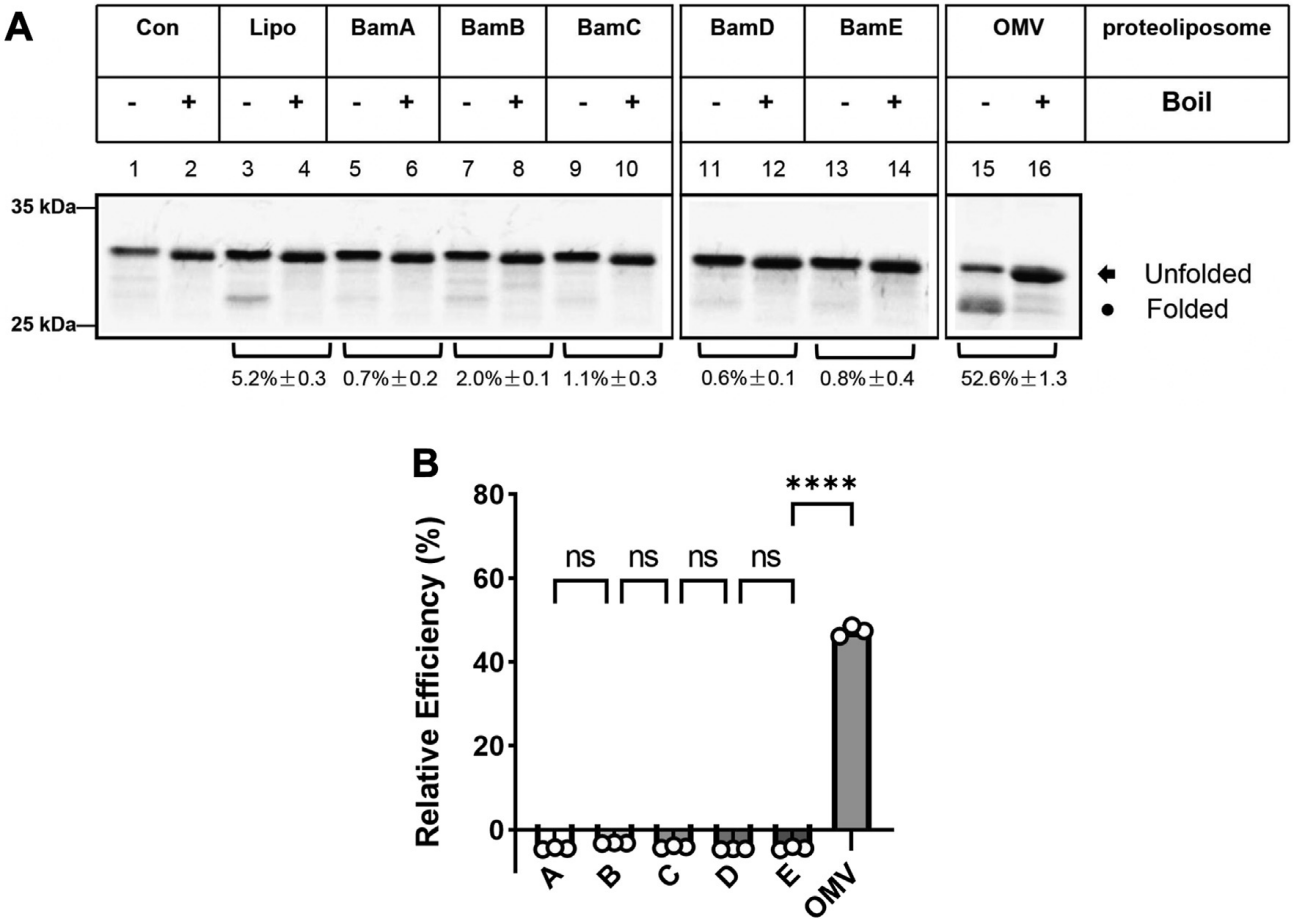
**Analysis of the assembly function of a single BAM component.***A*, spheroplasts over-expressing OmpA were mixed with INV buffer (lanes 1 and 2), liposomes (lanes 3 and 4) or proteoliposomes containing BamA (lanes 5 and 6), BamB (lanes 7 and 8), BamC (lanes 9 and 10), BamD (lanes 11 and 12), and BamE (lanes 13 and 14), or BAM-OMV (lanes 15 and 16). After incubation at 37 °C for 15 min, the reaction mixture was divided into two halves. One half was heated at 95 °C for 5 min, and the other half was treated for 15 min at 37 °C. All samples were separated by 12% SDS-PAGE, and visualized by a GE Typhoon Imager. Heat-modifiable bands of OmpA were marked as “Folded”. *B*, the relative assembly efficiency of a single BAM component was calculated by the amount of the density of observed folded bands versus that of liposomes using ImageQuant TL. All values are the averages of three independent measurements.

In addition, considering the relative stoichiometry of a functional *E. coli* BAM complex is likely to be 1:1:1:1:1 for each BAM protein (43), the defined-proteoliposomes used in the present work were prepared accordingly by the use of equal molar proteins to run through the reconstitution process as described (44). As a positive control, we also prepared proteoliposomes from outer membrane vesicles that contain overexpressed BAM complex (OMV-proteoliposomes) but before protein purification to guarantee that the handling of reconstitution and integration experiments is correct because we have shown that such proteoliposomes gave the best assembly activity (*cf.*, Fig. 2*A*, lane 15; Fig. 5*A*, compare lanes 15 and 17; and in other Figs. in lane OMV; and (42, 45)). To enable a relative comparison between the defined proteoliposomes and the best activity of BAM-OMV, Western blot analysis was performed to quantify the amount of BAM proteins embedded within both types of proteoliposomes. After a series of SDS-gel analysis and protein grayscale determinations (data now shown), the amount of BAM proteins contained in BAM-OMV and defined proteoliposomes was adjusted as equal (To conveniently compare the protein content, one-third of the above proteins were used for western-blot analysis, *i.e.*, 2.4 × 10^−5^ μmol). A typical western-blot analysis of the adjusted OMV- and defined-proteoliposomes is shown in Figure 1*D* using two essential BAM proteins (BamA and BamD) as representatives. The amount of each BAM component in the subsequent protein assembly experiment was kept equal, that is, 7.2 × 10^−5^ μmol.

**Figure 3:**
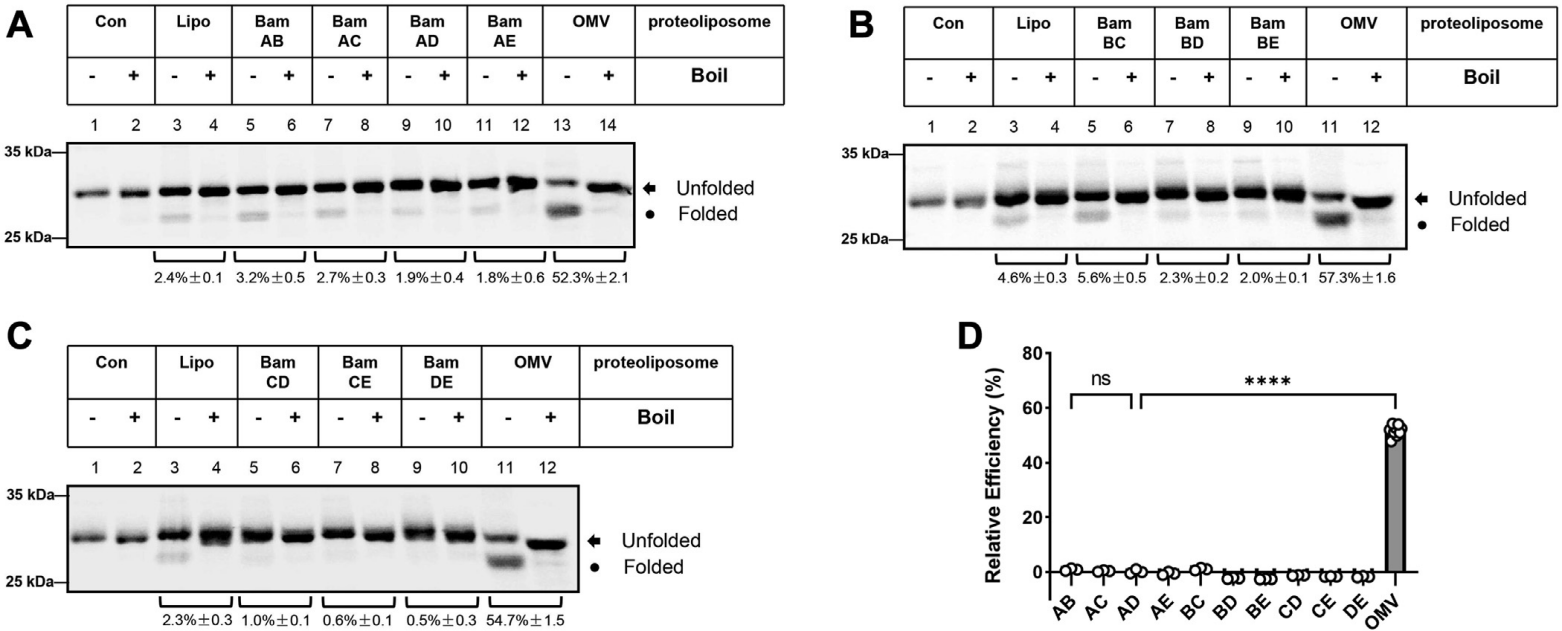
**Analysis of the assembly function of double BAM component combinations.***A*–*C*, double-combinations of the BAM components: BamA combined with BamB, BamC, BamD, and BamE, respectively; BamB combined with BamC, BamD, and BamE, respectively; as well as BamC combined with BamD, and BamE, respectively, were prepared into corresponding proteoliposomes. Spheroplasts-secreted OmpA were incubated with the above proteoliposomes, INV buffer, or liposomes and the following sample treatments of the reaction mixture are as described in Figure 2*A*. The representative images are shown in (*A*–*C*). *D*, the relative assembly efficiency of double BAM component combinations was calculated by the amount of the density of observed folded bands *versus* that of liposomes using ImageQuant TL. All values are the averages of three independent measurements.

**Figure 4:**
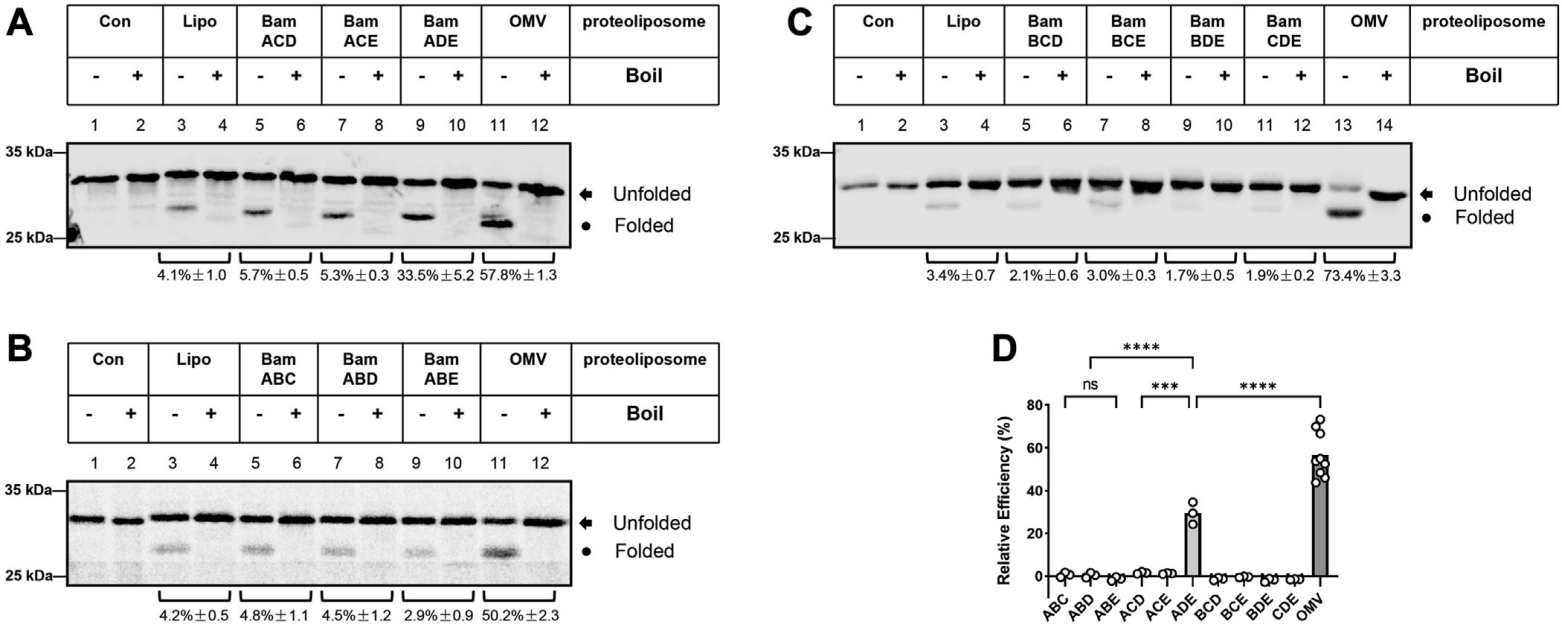
**BamADE are the core components of the *E. coli* BAM complex that form a minimum functional form to assemble OmpA.***A*, experiments with proteoliposomes containing triple BAM components combinations of BamACD, BamACE, and BamADE indicate that BamADE gave the most effective assembly of OmpA. The experiments conducted are as described in Figure 2*A*. *B*, Proteoliposomes containing triple BAM components combinations of BamABC, BamABD, and BamABE did not induce the folding of OmpA as compared with the Lipo group. *C*, the assembly of OmpA is almost impossible to occur in the absence of BamA in the case of triple combinations of BamBCD, BamBCE, BamBDE, and BamCDE. *D*, The relative assembly efficiency of triple BAM component combinations was calculated by the amount of the density of observed folded bands *versus* that of liposomes using ImageQuant TL. All values are the averages of three independent measurements.

**Figure 5:**
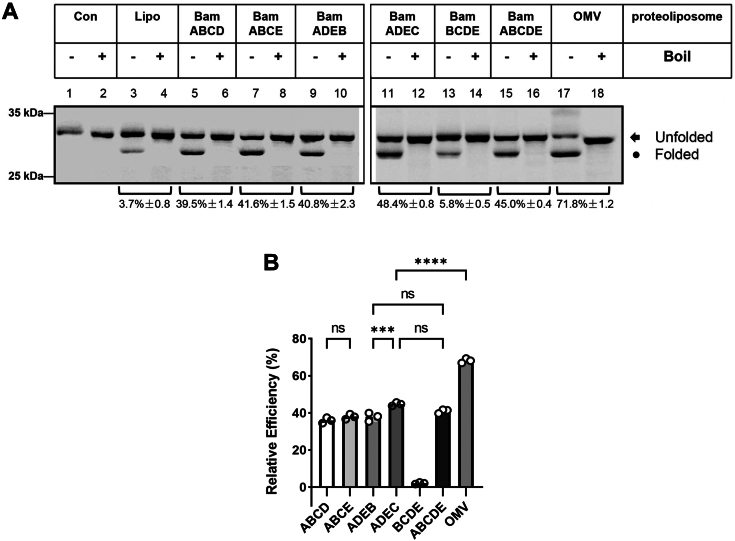
**Proteoliposomes containing four BAM components combinations (except BamBCDE) can effectively assemble OmpA.***A*, among the four-component- combinations, the assembly efficiency of BamADEC was the best, higher than that of BamADEB and other combinations, including the full BAM components combination, that is, BamABCDE. *B*, the relative assembly efficiency of four BAM components combinations was calculated by the amount of the density of observed folded bands versus that of liposomes using ImageQuant TL. All values are the averages of three independent measurements.

### Single or double combinations of the *E. coli* BAM component(s) show no assembly function toward OmpA

**Figure 6:**
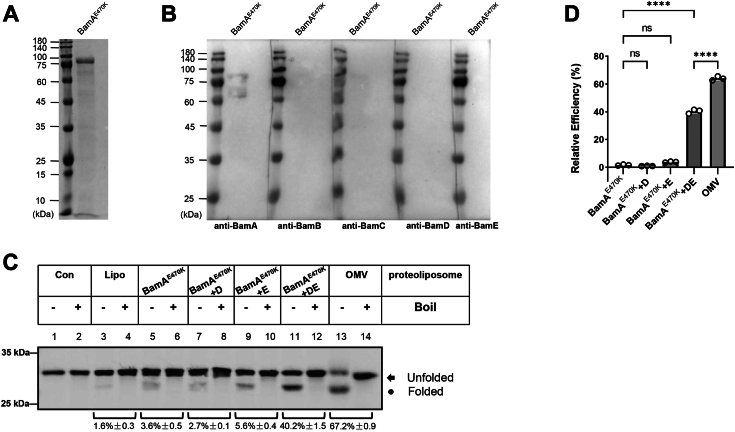
**BamA**^**E470K**^**mutant also requires BamD and BamE for an effective assembly of OmpA *in vitro*.***A*, 7.2 x 10^-5^ μmol BamA^E470K^ protein was loaded on a 10% SDS-PAGE gel and stained with Coomassie Blue R-250. The molecular weights of purified recombinant BamA^E470K^ protein is consistent with the theoretical sizes (BamA^E470K^ 94.1 kDa). *B*, the purity of BamA^E470K^ was detected by western-blot analysis using anti-BamA, BamB, BamC, BamD, and BamE antibodies. *C*, the proteoliposomes containing BamA^E470K^, BamA^E470K^+BamD, BamA^E470K^+BamE, and BamA^E470K^+BamD+BamE were mixed with spheroplasts-secreted OmpA, and then the assembly function was analyzed as described in Figure 2*A*. Clearly, BamA^E470K^ also requires BamD+BamE to effectively assemble OmpA (lane 11). *D*, the relative assembly efficiency was calculated by the amount of the density of observed folded bands *versus* that of liposomes using ImageQuant TL. All values are the averages of three independent measurements.

Outer membrane protein A (OmpA) is one of the predominant proteins found in the OM of *E. coli* and other Enterobacteriaceae that interacts specifically with the peptidoglycan layer to exert multiple functions (46, 47). OmpA comprises eight antiparallel β-strands and surprisingly tolerates extensive mutational alterations, which thus has been intensely used as a model substrate in the field of membrane protein folding (29, 41, 48, 49). Accumulated evidence suggests that the unfolded polypeptide chain of OmpA is capable of adopting its β-barrel structure after contacting with an amphiphilic entity and thus holds an intrinsic spontaneous folding ability (*cf.*, Figure 2, Figure 3, Figure 4, Figure 5, Figure 6, lane 3 and (46, 49, 50)). However, the folding process can be greatly accelerated in the presence of the BAM complex and chaperones (50, 51). In the present work, to mimic the *de novo* situation of the OmpA protein before its integration into the OM, we used again a cell-free spheroplast approach to recapitulate the *in vivo* biogenesis of newly synthesized OmpA (Fig. S1), because this approach faithfully reproduces the natural state of OMPs in an integration-competent state after their secretion by the Sec-translocon into the periplasm (42, 52). Spheroplast-secreted OmpA after induction, pulse-labeling with [^35^S]-methionine/cysteine and integration into OMV-proteoliposomes show a clear heat-modifiability (Fig. 2*A*, lanes 15 and 16; and (42)), which is a typical property for OMPs meaning that proteins maintain a compact structure when treated at low temperatures in the presence of SDS and thus show a faster-running behavior on polysaccharide gel (Fig. S1, “Folded”). As a result of boiling in SDS, the folded β-barrel OMPs become completely denatured and thus show a slower migration on polysaccharide gel compared with the folded version (Fig. S1). This heat modifiability is generally used as an indication of the successful folding of OMPs (42, 53, 54). No inherent folding property of the spheroplast-secreted OmpA was observed in our experimental set-up as demonstrated in Figure 2, Figure 3, Figure 4, Figure 5, Figure 6 (lanes 1 and 2) except in the presence of plain liposomes containing no proteins (Figure 2, Figure 3, Figure 4, Figure 5, Figure 6, lanes 3 and 4). However, the observed folding efficiency with plain liposomes had never exceeded 5.5% in our system, which we attributed to the inherent lipid-induced folding property of OmpA as described (46, 49, 50). When the essential BAM component BamA alone was reconstituted into proteoliposomes, no heat-modifiable band was observed (Fig. 2*A*, lanes 5 and 6) suggesting that BamA alone almost cannot assemble OmpA protein into the membranes. Similarly, proteoliposomes reconstituted from any single BamB-E component did not produce any heat-modifiable bands as well (Fig. 2*A*, lanes 7–12, and Fig. 2*B*), therefore further confirming that a single BAM component does not show any assembly function.

Considering individual genetic mutation or deletion of BamA and BamD in *E. coli* have been shown to affect the assembly of OMPs (24, 55), and disturbance of the coordination between BamA and BamD results in jamming of a lipoprotein RcsF on the BAM complex (56), we wondered the possibility that if BamA and BamD together can form a minimum functional unit. We next sought to construct proteoliposomes derived from double BAM components. As shown in Figure 3*A*, proteoliposomes reconstituted from the combination of BamA and BamD gave only a basal amount of heat-modifiable OmpA (Fig. 3*A*, lanes 9 and 10), which is comparable with that of liposomes (Fig. 3*A*, compare lanes 9 and 3) indicating that BamA and BamD together do not generate a significant amount of folded OmpA despite both being critical BAM components. The examination was further extended to the combination of BamA and BamB considering the regulatory function of BamB toward BamA (36), and the results (Fig. 3*A*, lanes 5 and 6) show that no obvious assembly function of BamA+BamB proteoliposomes toward OmpA was observed as well (Fig. 3*A*, compare lanes 5 and 3). Further combinations including BamA with BamC (Fig. 3*A*, lanes 7 and 8) or BamE (Fig. 3*A*, lanes 11 and 12) obtained the same results as BamA+BamD, that is, no assembly function was observed. Furthermore, similar results (Fig. 3*D*) were obtained in the case of other dual combinations (Fig. 3, *B* and *C*) thus demonstrating that none of the double BAM components could form a minimum functional unit to exert the assembly function.

### BamADE are the core components that form an effective minimum functional form to assemble OmpA

Based on the abovementioned results of double combinations of the BAM components, our examination was further extended to the combinations of 3 *E. coli* BAM components. Interestingly, proteoliposomes derived from the combination of BamADE result in a surprisingly obvious appearance of heat-modifiable bands (Fig. 4*A*, lanes 9 and 10) indicating the OmpA is successfully assembled, and the integration efficiency can reach up to 39%, which is almost 2/3rds compared with that of the BAM-OMV group, suggesting that a functional assembly unit was formed among BamADE. Moreover, proteoliposomes derived from the combinations of BamACD (Fig. 4*A*, lanes 5 and 6) and BamACE (Fig. 4*A*, lanes 7 and 8) seem to induce the formation of a certain amount of heat-modifiable OmpA, however, when compared with the control group in the presence of plain liposomes (Fig. 4*A*, lanes 3 and 4), the induction of the folded OmpA was not significant compared with that of BamADE (5.7 ± 0.5% and 5.3 ± 0.3% *versus* 33.5 ± 5.2%). Furthermore, it is interesting to mention that proteoliposomes prepared from the combinations of BamABC/ABD/ABE (Fig. 4*B*, lanes 5–10) and combinations without BamA (*i.e.*, BamBCD/BCE/BDE/CDE, Fig. 4*C*) gave hardly any heat-modifiable bands of OmpA, which therefore *vice verse* strongly suggest that BamADE-induced OmpA integration was indeed a functional event of the BamADE components (Fig. 4*D*). Altogether, these results thus demonstrate that the successful assembly of OmpA requires also BamE beside BamA and BamD, and thus BamADE can be regarded as the core components of the *E. coli* BAM complex that form a minimum and effective functional unit.

### The redundancy between BamB and BamC as well as BamBC and BamD or BamE

Although the *bamB* gene is ubiquitously distributed in the genomes of α-, β-, and γ-proteobacteria (5), *ΔbamB* mutant does not affect cell growth (35) despite a regulatory function toward BamA was suggested (36). Similarly, the knockout of *bamC* does not lead to any distinctive phenotype (35). Since the abovementioned results demonstrated that BamADE functions as the minimum form to assemble OmpA, it would be interesting to distinguish and compare how BamB and BamC affect the assembly of OmpA by including BamB and BamC respectively into this minimum form. To this end, we conducted experiments from the combinations of 4 *E. coli* BAM components with BamADE+BamB and BamADE+BamC. As expected, the heat-modifiable form of OmpA was observed in both combinations (Fig. 5*A*, lanes 9–12); however, when the assembly efficiency was calculated from at least three independent experiments, BamADEC (48.4 ± 0.8%) gave a better assembly efficiency than that of BamADEB (40.8 ± 2.3%) when both compared with that of BamADE (33.5 ± 5.2%), suggesting that BamC has a better assembly promoting function than that of BamB.

A further test by use of both BamB and BamC together with BamADE minimum form (*i.e.*, BamADEBC, the full BAM complex, Figure 5*A*, lanes 15 and 16) shows that the obtained assembly efficiency was about 45% (Figure 5*A*, lanes 15 and 16), which is even lower than that of BamADEC (48%). Moreover, experiments with these full BAM proteoliposomes indicate that all the components purified in the present work possess their functionality (Fig. 5*B*). We further asked how the other four-component combinations affect the assembly of OmpA and for this purpose, proteoliposomes containing BamABCD and BamABCE were reconstituted and subjected to the assembly experiments toward OmpA. As shown in Figure 5*A* (lanes 5–8), clearly both four-component combinations lead to the formation of heat-modifiable OmpA bands therefore suggesting that the combinations of four BAM components are functional except in the absence of BamA (*i.e.*, BamBCDE, in Figure 5*A*, lanes 13 and 14).

### *E. coli* BamA^E470K^ mutant also requires BamD and BamE to assemble OmpA *in vitro*

Genetic suppression analyses have identified a functional mutant of BamA (BamA^E470K^) that is hypothesized to bypass the functional requirement of BamD and other BAM lipoproteins (33, 39). We are curious to ask whether the BamA^E470K^ mutant protein alone can assemble OmpA or not *in vitro*. To this end, we over-expressed and purified BamA^E470K^ protein (Fig. 6*A*), and used western-blot analysis to confirm the identity and purity of the purified proteins. As shown in Figure 6*B*, no residual contaminations from BamB/BamC/BamD/BamE were observed therefore any potential effects from cross-reaction of other BAM lipoproteins with BamA^E470K^ can be excluded. The purified BamA^E470K^ proteins were subjected to the reconstitution process to prepare proteoliposomes containing BamA^E470K^, BamA^E470K^+BamD, BamA^E470K^+BamE, and BamA^E470K^+BamD+BamE. No heat-modifiable OmpA band was observed with proteoliposomes containing only BamA^E470K^ (Fig. 6*C*, lanes 5 and 6), indicating that BamA^E470K^ alone is not sufficient to assemble OmpA. Further supplementation of BamD or BamE to BamA^E470K^ did not promote the assembly function of BamA^E470K^ toward OmpA as well (Fig. 6*C*, lanes 7–10). However, in sharp contrast, proteoliposomes containing BamA^E470K^+BamD+BamE gave rise to apparently the formation of heat-modifiable OmpA bands thus demonstrating that OmpA is now successfully inserted into the membranes (Fig. 6*C*, compare lane 11 and lane 3, as well as lanes 5, 7, and 9), and the calculated assembly efficiency can reach about 40%, which is almost 3/5ths compared with that of the BAM-OMV proteoliposomes (Fig. 6*C*, lanes 11–14 and Fig. 6*D*).

### The BamADE minimum form is also able to assemble large OMPs, that is, BamA

**Figure 7:**
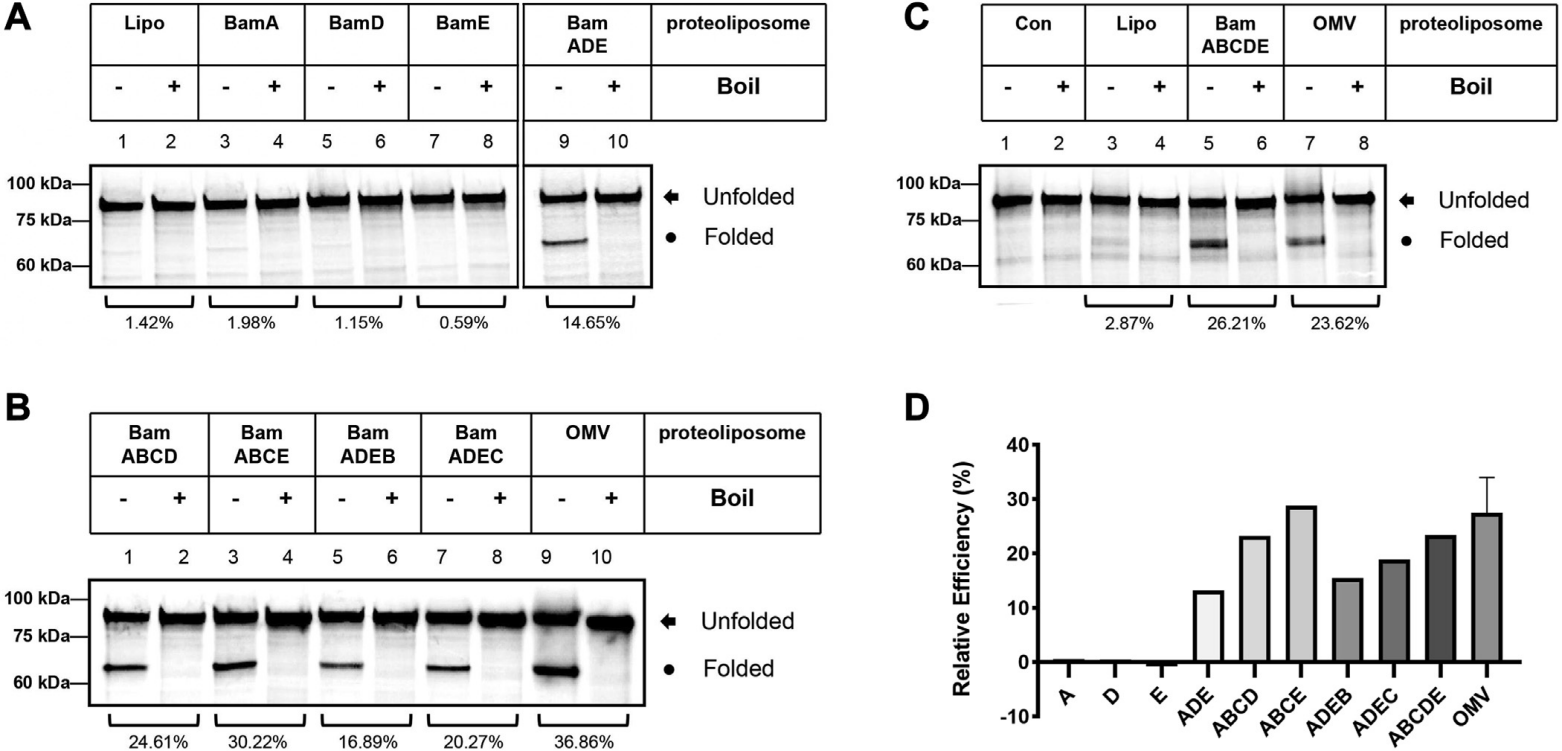
**Assembly of 16 β-stranded BamA protein.***A*, while any single BamA, BamD, or BamE component does not enable the formation of heat-modifiable bands of BamA, proteoliposomes containing BamADE lead to the appearance of heat-modifiable BamA bands on semi-native SDS gel. *B*, four-component combinations of BAM proteins are effective in the assembly of BamA as indicated by the formation of heat-modifiable bands. *C*, the assembly of BamA in the presence of INV, plain liposomes, proteoliposomes containing BamABCDE proteins, and proteoliposomes prepared from outer membranes (OMV) over-expressing the BAM complex. *D*, the relative assembly efficiency was calculated by the amount of the density of observed folded bands *versus* that of liposomes using ImageQuant TL.

Recently, Thewasano *et al.* (29) categorized the OMP substrates according to their requirement of the auxiliary BamB-E proteins and concluded that BamB is required for the efficient assembly of OMPs containing 16 or more β-strands but not for those OMPs less than 12 β-strands, and BamC seems to be not required for the efficient assembly of all OMPs regardless of their sizes. To further examine whether the observed effects on OmpA are only limited to small-sized OMPs (*i.e.*, 8 β-stranded OmpA) or hold the same case on large OMPs, we performed the experiments using BamA protein (16 β-strands) as an integration substrate. As shown in Figure 7, spheroplast-secreted BamA lacks the heat-modifiable property in the presence of plain liposomes (Fig. 7*A*, lanes 1 and 2 and Fig. 7*C*, lanes 3 and 4) suggesting that unlike OmpA protein, an inherent lipid-induced folding ability does not apply to BamA protein. Moreover, considering BamD was previously shown to bind to the unfolded BamA and is alone able to assemble BamA into the membranes (57), we performed the integration experiments using proteoliposomes prepared from each of the extensively purified BamA, BamD, or BamE protein and as shown in Figure 7*A* (lanes 3–8), no heat-modifiable bands of BamA were observed thus demonstrating that no assembly activity toward BamA from any single BamA, BamD, or BamE could be obtained under the current experimental conditions. In sharp contrast, proteoliposomes prepared from the combinations of BamA, BamD, and BamE (*i.e.*, BamADE in Fig. 7*A*, lanes 9 and 10) gave rise to a clear appearance of heat-modifiable BamA bands (Fig. 7*A*, lanes 9, “Folded”), thus suggesting that the BamADE constituted minimum form is functionally able to assemble large OMPs into the membranes as well.

To further understand the functional role of *E. coli* BAM components in the assembly of large OMPs, four-component combinations were conducted. As shown in Figure 7*B*, proteoliposomes prepared from the combinations of BamADE+BamB and BamADE+BamC both enabled a better assembly of BamA (compare Fig. 7*B*, lanes 5–8 with Fig. 7*A*, lanes 9 and 10) compared with that of the BamADE minimum form, and it is obvious that BamC has a better stimulation effect than that of BamB (Fig. 7*D*), which thus further confirms that BamC has a better assembly promoting function than that of BamB. Interestingly, four-component combinations of BamABCD and BamABCE also showed an efficient assembly function toward BamA (Fig. 7*B*, lanes 1–4), therefore further confirming the results observed with that of OmpA. Finally, the proteoliposomes containing the intact BAM complex, that is, the five purified proteins BamA-E, achieved a similar assembly efficiency as that of the four-component-combinations (Fig. 7*D*) indicating that indeed a functional redundancy among the auxiliary BAM components does exist.

## Discussion

**Figure 8:**
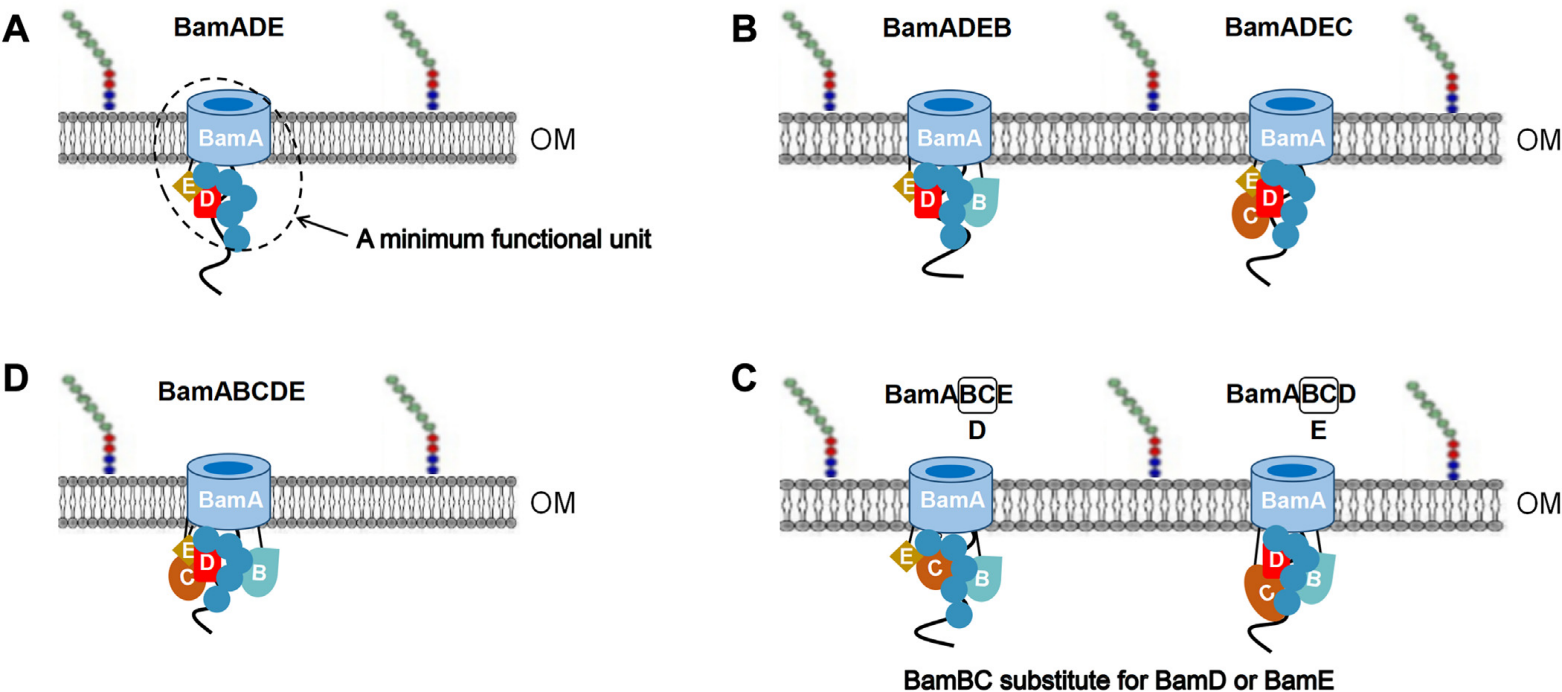
**Summary of the results.***A*, BamADE constitutes the effective minimum functional form of the *E. coli* BAM complex to assemble OMPs. *B*, based on the BamADE minimum form, both BamB and BamC promote the assembly efficiency of OMPs. *C*, BamB and BamC have a redundant function in the assembly of OMPs, and both together could cooperate with each other to substitute for the function of BamD or BamE, respectively. *D*, the intact BAM complex composed of BamA-E five proteins achieved the correct assembly of OMPs.

Considering a biochemically defined method holds the advantage of directly addressing the function of individual BAM components, and the observed effects are a direct consequence of the included component (58), in the present work, we sought to use the reconstitution strategy to examine the minimum functional form of the *E. coli* BAM complex and to investigate the redundancy of each BAM component. Our results indicate that any single or double combinations of the BAM components cannot assemble OmpA or BamA and only BamADE among the three-component combinations can successfully assemble both proteins into the membranes (Fig. 8*A*). Interestingly, the mutant BamA^E470K^, which is believed to bypass the requirement of BamD and other BAM lipoproteins *in vivo*, assembles OmpA only in the presence of BamD and BamE, which *vice verse* suggests that the observed requirement of BamD and BamE is a functional necessity at least *in vitro*. This observation is not surprising because although BamA^E470K^ seemingly can assemble certain levels of OMPs to maintain a certain degree of cell viability in the absence of BamBCDE under *in vivo* conditions, the OMP levels were strongly reduced (33) indicating that the assembly function of BamA^E470K^ alone in the absence of BamDE is severely defective, and the possibility that other yet-to-be characterized outer envelope components that may support the function of BamA^E470K^ in cells cannot be excluded. On the same line, BamE was not only recently demonstrated to interact directly with both BamA and BamD and plays an essential role particularly when BamA/BamD communication is impeded (34) but also was required in assisting seven different barrel-forming proteins (29). Therefore, together with our biochemical reconstitution data, it is tempting to conclude that BamADE is the minimum functional form for the effective assembly of OMPs by the *E. coli* BAM complex (Fig. 8*A*). However, it should be noted that the present work tested only two representative proteins and in the future more OMPs should be examined to generalize this observation considering that *ΔbamB ΔbamC* double mutant showed a temperature sensitivity (33), suggesting that under certain conditions BamADE might not always be sufficient to assemble all OMPs.

While collectively essential for cell viability, individual genetic deletion of *bamB*, *bamC*, or *bamE* is dispensable for *E. coli* cells (33, 35), which makes it difficult to analyze the individual function of each BamBCE component using genetic strategy. In contrast, the reconstitution method is now able to provide a functional comparison among individual BAM components based on the minimum functional form of the *E. coli* BAM complex, that is, BamADE. It is obvious that both BamB and BamC promote the assembly of OmpA and BamA, but BamC has a better stimulation effect than BamB toward the assembly of both proteins based on the function of BamADE (Fig. 8*B*). Although the stimulation effect of BamB has been recorded (59), it is surprising to us that BamC has a better effect than BamB because no strong phenotype of *ΔbamC* was recorded, which likely suggests a less important role of BamC. In contrast, knock out of the *bamB* gene leads to the impairment of cell viability, increase of OM permeability, and decrease of OMP content, and the *bamB* mutant showed a strong fitness defect (60, 61), therefore indicating that BamB might play an important role in bacterial cell. Clearly, further extensive investigations to understand the stimulation effect of BamC observed in the present work are required to reveal the exact function of this mystery component.

Moreover, a further combination of BamBC together (*i.e.*, BamADEBC, Fig. 8*D*) in OmpA and BamA assembly did not give a combined stimulation effect than BamB or BamC alone (Fig. 5*B*), indicating that BamB and BamC might have a redundant function in the assembly of OMPs. More interestingly, other four-component combinations show that in the presence of BamB and BamC, the lack of core components BamD (*i.e.*, BamABCE) or BamE (i.e. BamABCD) did not cause the loss of assembly activity of the rest of BAM components. By assuming that BamB and BamC cooperate with each other to functionally substitute for the missing component of BamD or BamE, respectively, that is, BamABCE corresponds to BamADE (Fig. 8*C*, *left*) and BamABCD corresponds to BamAED (Fig. 8*C*, *right*), the results (Figs. 5*A* and 8C) are easily understandable as this corresponds to the situation that the intactness of BamADE is maintained. Furthermore, it is also easy to understand why *ΔbamB ΔbamC* mutant (correlates with BamADE) only showed slight temperature sensitivity, while *ΔbamB ΔbamE* mutant (lack of BamB means the cooperation of BamBC was destroyed, which is like the missing function of BamD, therefore together with *ΔbamE*, it is like the lack of BamDE) exhibited a significant conditional lethality (33). Furthermore, the lethality of the triple mutant *ΔbamB ΔbamC ΔbamE* is also understandable because the mutant is like the lack of BamDE from the BamADE minimum form. However, considering crystal structures in the absence of an integration substrate see only contact of BamC to the BAM complex *via* BamE, and no direct contact of BamC to BamB was recorded (62, 63), therefore it should be noted that whether this cooperation between BamB and BamC requires physical interaction, and if so, how this interaction occurs and if it only exists when BamD or BamE is lacking within the BAM subcomplex containing four BAM components, or occurs also within the BAM complex containing five BAM components, requiring further extensive investigations.

The identification of the three components composed effective minimum functional form of the BAM complex in *E. coli* is not surprising because not only BamA and BamD are deemed essential in OMP assembly, BamE was also shown to stabilize the BAM complex and its deletion affects OMP assembly (29). Moreover, evolutionally bacterial-derived eukaryotic organelles mitochondria and chloroplasts both contain three components comprised of SAM complex and TOC complex in their OM, respectively (13, 64, 65, 66, 67), which is indicative of the conservation of overall important structural construction (*i.e.*, three components) during evolution despite that no BamD and BamE homologous proteins have been found. Furthermore, the minimum functional form of BamADE suggests that future mechanistic investigations should also include BAM lipoproteins considering that the current investigations mainly focus on BamA.

## Experimental procedures

### Bacterial strains and plasmids

*E. coli* BL21 (DE3) strains containing plasmids pTrc99a::OmpA or expressing all BAM complex subunits were stored in our lab as described in Ref. (42). The plasmid expressing BamA, pTrc99a::BamA, whose own signal peptide was replaced by the signal peptide of OmpA to improve the expression level of BamA, was constructed by DNA assembly kit (TransGen Biotech, China). The PCR fragment containing *bamA*, *bamA*^*E470K*^, *bamB*, *bamC*, *bamD*, or *bamE* gene was cloned into pET22b by DNA assembly kit, verified by DNA sequencing, and then transformed into *E. coli* BL21 (DE3). Plasmids pET22b::BamA and pET22b::BamA^E470K^ each contained DNA sequences encoding Strep-tag II (WSHPQFEK) at the gene C-terminal end, while plasmids pET22b::BamB, C, D, or E each has hexahistidine sequences (His_6_-tag). Primers used in this study were summarized in Table S1.

### Purification of recombinant proteins

*E. coli* BL21 (DE3) strains expressing single BAM subunit (BamA/BamA^E470K^/B/C/D/E) were cultured in LB medium at 37 °C, supplemented with 100 μg/ml Ampicillin. When optical density at 600 nm reached about 1.0, 0.2 mM isopropyl beta-D-thiogalacyranoside (IPTG) was added, and induced at 20 °C for about 16 h. The cells were then harvested by centrifugation, resuspended in buffer 1 (20 mM Tris HCl [pH 8.0], 150 mM NaCl), lysed through the French pressure at 8000 psi, and centrifuged at 18,000 r.p.m. for 1 h according to the Ref. (45). And then the supernatants obtained here were dissolved in buffer 2 (20 mM Tris HCl [pH 8.0], 300 mM NaCl, 1% n-Dodecyl-β-D-maltoside (DDM)) on ice for 2 h, followed by centrifugation at 18,000 r.p.m. at 4 °C for 1 h. For BamA or BamA^E470K^, which carries Strep-tag II, the supernatants in this step were loaded onto a StrepTrap XT column (Cytiva, 5 ml) in buffer A1 (20 mM Tris HCl [pH 8.0], 150 mM NaCl, 0.1% DDM), and then eluted with buffer B1 (20 mM Tris HCl [pH 8.0], 150 mM NaCl, 50 mM biotin, 0.1% DDM). For recombinant BamB, C, D, or E, which carry His_6_-tag, the supernatants were loaded onto a HisTrap HP column (Cytiva, 5 ml) pre-equilibrated with buffer A2 (20 mM Tris HCl [pH 8.0], 150 mM NaCl, 0.025% DDM), then eluted gradually with buffer B2 (20 mM Tris HCl [pH 8.0], 150 mM NaCl, 500 mM imidazole, 0.025% DDM). Afterward, the fractions were analyzed using 10%, 12%, or 15% SDS-PAGE based on the molecular weights of the proteins, and the fractions containing corresponding proteins were pooled and concentrated. The concentrated fractions were separately loaded onto a HiTrap Q HP column (Cytiva, 1 ml) in buffer A3 (20 mM Tris HCl [pH 8.8], 50 mM NaCl, 1 mM EDTA [pH 8.0], 0.1% DDM), and eluted with buffer B3 (20 mM Tris HCl [pH 8.8], 1 M NaCl, 1 mM EDTA [pH 8.0], 0.1% DDM). Then, the samples were loaded onto a Superdex G200 column (10/300; GE), and eluted with buffer C1 (20 mM Tris HCl [pH 8.0], 150 mM NaCl, 0.05% DDM). Fractions were analyzed by SDS-PAGE and the protein concentrations were determined by the Lowry method (68).

### Reconstitution of proteoliposomes

As for the proteoliposomes containing purified proteins, the procedure was conducted as described in Ref. (42). BamA, BamA^E470K^, B, C, D, or E protein was added in different combinations, each at the concentration of 6.25 μM. As for the reconstitution of BAM complex (BAM-OMV), first, BAM outer membrane vesicles were prepared according to the Refs. (42, 69), and then 10 μl outer membrane vesicles were solubilized at room temperature for 2 h in 150 μl PE buffer (20 mM Na_2_HPO_4_, 1% Elugent) by end-over-end rotation, followed by an ultra-centrifugation at 45,000 r.p.m. for 1 h at 4 °C to remove insoluble materials, the supernatants were mixed with 120 μl of 20 mM Na_2_HPO_4_ and 80 μl of 4 mg/ml Avanti *E. coli* phospholipids (Avanti). The above mixture was added to a SafeSeal Microcentrifuge tube (BioScience) containing 50 to 65 mg Biobeads SM-2 Adsorbent, 20 to 50 mesh (Bio-Rad) for three times, the details were described in Ref. (11).

### *In vitro* integration assay

The integration assay of OmpA was performed according to Ref. (42). In short, *E. coli* BL21 (DE3) strain over-expressing OmpA was prepared as spheroplasts, adjusted to a final OD_580_ of 4.0, and the experiment was performed in a post-secretion manner, in which the spheroplasts were first incubated at 37 °C for 15 min, then induced with 4 mM IPTG for OmpA expression, and then pulse-labeled at 37 °C with [^35^S]-EasyTag Express Protein Labeling Mix (PerkinElmer). After centrifugation at 13,000 r.p.m. for 6 min, 20 μl spheroplasts were mixed with liposomes or different proteoliposomes from different combinations of BamA, BamA^E470K^, B, C, D, or E (or BAM-OMV) at 37 °C for 15 min, and then the supernatants were divided into two halves. One half was heated at 95 °C for 5 min, and the other half was treated for 15 min at 37 °C.

The integration assay of BamA was also performed according to Ref. (42) in a co-secretion manner with a minor modification. After incubation with different proteoliposomes at 37 °C for 15 min, the mixture was centrifugated at 13,000 r.p.m. for 6 min, and then the supernatants were divided into two halves. One half was heated at 95 °C for 5 min, and the other half was kept at 18 °C without shaking. The samples of OmpA were separated by 12% SDS-PAGE, while BamA samples were analyzed by 4 to 12% SurePAGE (Genscript Biotech), and visualized on GE Typhoon Imager. To calculate the relative efficiency, the ^35^S-labeled protein bands were first quantified using ImageQuant TL based on the band intensity, and then the assembly efficiency was calculated as the ratio of quantification from folded bands divided by the sum of folded and unfolded ones. The “relative efficiency” of each group was calculated by subtracting the assembly efficiency of the corresponding lipo group, the data shown in the Figures were from three independent experiments. The error bars shown in Figure 2, Figure 3, Figure 4, Figure 5*B* and 6C represented the standard deviation of data in three independent experiments. Data were performed one-way ANOVA by using Graphpad Prism 9.0 software, expressed as mean ± standard deviation (mean ± SD, n = 3), ns represents not significant, ∗ means *p* < 0.05, ∗ ∗ means *p* < 0.01, ∗ ∗ ∗ means *p* < 0.001, and ∗ ∗ ∗ ∗ means *p* < 0.0001.

## Data availability

The manuscript contains all the data.

## Supporting information

This article contains supporting information (42, 44).

## Conflict of interest

The authors declare that they have no known competing financial interests or personal relationships that could have appeared to influence the work reported in this paper.

## References

[1] Nikaido H., Nakae T. (1979). The outer membrane of Gram-negative bacteria. Adv. Microb. Physiol..

[2] Klebba P.E., Newton S.M. (1998). Mechanisms of solute transport through outer membrane porins: burning down the house. Curr. Opin. Microbiol..

[3] Rapoport T.A. (2007). Protein translocation across the eukaryotic endoplasmic reticulum and bacterial plasma membranes. Nature.

[4] Ranava D., Caumont-Sarcos A., Albenne C., Ieva R. (2018). Bacterial machineries for the assembly of membrane-embedded β-barrel proteins. FEMS Microbiol. Lett..

[5] Webb C.T., Heinz E., Lithgow T. (2012). Evolution of the β-barrel assembly machinery. Trends Microbiol..

[6] Heinz E., Lithgow T. (2014). A comprehensive analysis of the Omp85/TpsB protein superfamily structural diversity, taxonomic occurrence, and evolution. Front. Microbiol..

[7] Hagan C.L., Silhavy T.J., Kahne D. (2011). β-barrel membrane protein assembly by the Bam complex. Annu. Rev. Biochem..

[8] Volokhina E.B., Beckers F., Tommassen J., Bos M.P. (2009). The beta-barrel outer membrane protein assembly complex of *Neisseria meningitidis*. J. Bacteriol..

[9] Dunn J.P., Kenedy M.R., Iqbal H., Akins D.R. (2015). Characterization of the β-barrel assembly machine accessory lipoproteins from *Borrelia burgdorferi*. BMC Microbiol..

[10] Nesper J., Brosig A., Ringler P., Patel G.J., Müller S.A., Kleinschmidt J.H. (2008). Omp85(*Tt*) from *Thermus thermophilus* HB27: an ancestral type of the Omp85 protein family. J. Bacteriol..

[11] Estrada Mallarino L., Fan E., Odermatt M., Müller M., Lin M., Liang J. (2015). *Tt*Omp85, a β-barrel assembly protein, functions by barrel augmentation. Biochemistry.

[12] Wiedemann N., Kozjak V., Chacinska A., Schönfisch B., Rospert S., Ryan M.T. (2003). Machinery for protein sorting and assembly in the mitochondrial outer membrane. Nature.

[13] Paschen S.A., Waizenegger T., Stan T., Preuss M., Cyrklaff M., Hell K. (2003). Evolutionary conservation of biogenesis of beta-barrel membrane proteins. Nature.

[14] Klein A., Israel L., Lackey S.W., Nargang F.E., Imhof A., Baumeister W. (2012). Characterization of the insertase for β-barrel proteins of the outer mitochondrial membrane. J. Cell Biol..

[15] Schleiff E., Soll J., Küchler M., Kühlbrandt W., Harrer R. (2003). Characterization of the translocon of the outer envelope of chloroplasts. J. Cell Biol..

[16] Reumann S., Davila-Aponte J., Keegstra K. (1999). The evolutionary origin of the protein-translocating channel of chloroplastic envelope membranes: identification of a cyanobacterial homolog. Proc. Natl. Acad. Sci. U. S. A..

[17] Voulhoux R., Bos M.P., Geurtsen J., Mols M., Tommassen J. (2003). Role of a highly conserved bacterial protein in outer membrane protein assembly. Science.

[18] Doerner P.A., Sousa M.C. (2015). Small angle X-ray scattering (SAXS) characterization of the POTRA domains of BamA. Methods Mol. Biol..

[19] Arnold T., Zeth K., Linke D. (2010). Omp85 from the *thermophilic cyanobacterium Thermosynechococcus elongatus* differs from proteobacterial Omp85 in structure and domain composition. J. Biol. Chem..

[20] Doyle M.T., Jimah J.R., Dowdy T., Ohlemacher S.I., Larion M., Hinshaw J.E. (2022). Cryo-EM structures reveal multiple stages of bacterial outer membrane protein folding. Cell.

[21] Lee J., Tomasek D., Santos T.M., May M.D., Meuskens I., Kahne D. (2019). Formation of a β-barrel membrane protein is catalyzed by the interior surface of the assembly machine protein BamA. Elife.

[22] Shen C., Chang S., Luo Q., Chan K.C., Zhang Z., Luo B. (2023). Structural basis of BAM-mediated outer membrane β-barrel protein assembly. Nature.

[23] Wu R., Bakelar J.W., Lundquist K., Zhang Z., Kuo K.M., Ryoo D. (2021). Plasticity within the barrel domain of BamA mediates a hybrid-barrel mechanism by BAM. Nat. Commun..

[24] Malinverni J.C., Werner J., Kim S., Sklar J.G., Kahne D., Misra R. (2006). YfiO stabilizes the YaeT complex and is essential for outer membrane protein assembly in *Escherichia coli*. Mol. Microbiol..

[25] Anwari K., Webb C.T., Poggio S., Perry A.J., Belousoff M., Celik N. (2012). The evolution of new lipoprotein subunits of the bacterial outer membrane BAM complex. Mol. Microbiol..

[26] Bos M.P., Robert V., Tommassen J. (2007). Biogenesis of the Gram-negative bacterial outer membrane. Annu. Rev. Microbiol..

[27] Gatsos X., Perry A.J., Anwari K., Dolezal P., Wolynec P.P., Likić V.A. (2008). Protein secretion and outer membrane assembly in Alphaproteobacteria. FEMS Microbiol. Rev..

[28] Kim S., Malinverni J.C., Sliz P., Silhavy T.J., Harrison S.C., Kahne D. (2007). Structure and function of an essential component of the outer membrane protein assembly machine. Science.

[29] Thewasano N., Germany E.M., Maruno Y., Nakajima Y., Shiota T. (2023). Categorization of *Escherichia coli* outer membrane proteins by dependence on accessory proteins of the β-barrel assembly machinery complex. J. Biol. Chem..

[30] Lee J., Xue M., Wzorek J.S., Wu T., Grabowicz M., Gronenberg L.S. (2016). Characterization of a stalled complex on the β-barrel assembly machine. Proc. Natl. Acad. Sci. U. S. A..

[31] Lee J., Sutterlin H.A., Wzorek J.S., Mandler M.D., Hagan C.L., Grabowicz M. (2018). Substrate binding to BamD triggers a conformational change in BamA to control membrane insertion. Proc. Natl. Acad. Sci. U. S. A..

[32] Diederichs K.A., Buchanan S.K., Botos I. (2021). Building better barrels - β-barrel biogenesis and insertion in bacteria and mitochondria. J. Mol. Biol..

[33] Hart E.M., Silhavy T.J. (2020). Functions of the BamBCDE lipoproteins revealed by bypass mutations in BamA. J. Bacteriol..

[34] Kumar S., Konovalova A. (2023). BamE directly interacts with BamA and BamD coordinating their functions. Mol. Microbiol..

[35] Rigel N.W., Schwalm J., Ricci D.P., Silhavy T.J. (2012). BamE modulates the *Escherichia coli* beta-barrel assembly machine component BamA. J. Bacteriol..

[36] Chen Z., Zhan L.H., Hou H.F., Gao Z.Q., Xu J.H., Dong C. (2016). Structural basis for the interaction of BamB with the POTRA3-4 domains of BamA. Acta Crystallogr. D Struct. Biol..

[37] McCabe A.L., Ricci D., Adetunji M., Silhavy T.J. (2017). Conformational changes that coordinate the activity of BamA and BamD allowing β-barrel assembly. J. Bacteriol..

[38] Ricci D.P., Hagan C.L., Kahne D., Silhavy T.J. (2012). Activation of the *Escherichia coli* β-barrel assembly machine (BAM) is required for essential components to interact properly with substrate. Proc. Natl. Acad. Sci. U. S. A..

[39] Hart E.M., Gupta M., Wühr M., Silhavy T.J. (2020). The gain-of-function allele bamA(E470K) bypasses the essential requirement for BamD in β-barrel outer membrane protein assembly. Proc. Natl. Acad. Sci. U. S. A..

[40] Tellez R., Misra R. (2012). Substitutions in the BamA β-barrel domain overcome the conditional lethal phenotype of a *ΔbamB ΔbamE* strain of *Escherichia coli*. J. Bacteriol..

[41] Chu Y., Wang Z., Weigold S., Norrell D., Fan E. (2021). *Tt*Omp85, a single Omp85 member protein functions as a β-barrel protein insertase and an autotransporter translocase without any accessory proteins. Biochem. Biophys. Res. Commun..

[42] Norell D., Heuck A., Tran-Thi T.A., Götzke H., Jacob-Dubuisson F., Clausen T. (2014). Versatile *in vitro* system to study translocation and functional integration of bacterial outer membrane proteins. Nat. Commun..

[43] Hagan C.L., Kim S., Kahne D. (2010). Reconstitution of outer membrane protein assembly from purified components. Science.

[44] Fan E., Norell D., Müller M. (2015). An *in vitro* assay for substrate translocation by FhaC in liposomes. Methods Mol. Biol..

[45] Fan E., Fiedler S., Jacob-Dubuisson F., Müller M. (2012). Two-partner secretion of gram-negative bacteria: a single β-barrel protein enables transport across the outer membrane. J. Biol. Chem..

[46] Koebnik R., Locher K.P., Van Gelder P. (2000). Structure and function of bacterial outer membrane proteins: barrels in a nutshell. Mol. Microbiol..

[47] Nie D., Hu Y., Chen Z., Li M., Hou Z., Luo X. (2020). Outer membrane protein A (OmpA) as a potential therapeutic target for *Acinetobacter baumannii* infection. J. Biomed. Sci..

[48] Hussain S., Bernstein H.D. (2018). The BAM complex catalyzes efficient insertion of bacterial outer membrane proteins into membrane vesicles of variable lipid composition. J. Biol. Chem..

[49] Kleinschmidt J.H. (2003). Membrane protein folding on the example of outer membrane protein A of *Escherichia coli*. Cell. Mol. Life Sci..

[50] Gessmann D., Chung Y.H., Danoff E.J., Plummer A.M., Sandlin C.W., Zaccai N.R. (2014). Outer membrane β-barrel protein folding is physically controlled by periplasmic lipid head groups and BamA. Proc. Natl. Acad. Sci. U. S. A..

[51] Bulieris P.V., Behrens S., Holst O., Kleinschmidt J.H. (2003). Folding and insertion of the outer membrane protein OmpA is assisted by the chaperone Skp and by lipopolysaccharide. J. Biol. Chem..

[52] Tokuda H. (2009). Biogenesis of outer membranes in Gram-negative bacteria. Biosci. Biotechnol. Biochem..

[53] Nakamura K., Mizushima S. (1976). Effects of heating in dodecyl sulfate solution on the conformation and electrophoretic mobility of isolated major outer membrane proteins from *Escherichia coli* K-12. J. Biochem..

[54] Tommassen J. (2010). Assembly of outer-membrane proteins in bacteria and mitochondria. Microbiology (Reading).

[55] Doerrler W.T., Raetz C.R. (2005). Loss of outer membrane proteins without inhibition of lipid export in an *Escherichia coli* YaeT mutant. J. Biol. Chem..

[56] Tata M., Konovalova A. (2019). Improper coordination of BamA and BamD results in BAM complex jamming by a lipoprotein substrate. mBio.

[57] Hagan C.L., Westwood D.B., Kahne D. (2013). Bam lipoproteins assemble BamA *in vitro*. Biochemistry.

[58] Kuhn A. (2019). Crosslinking and reconstitution approaches to study protein transport. Protein J..

[59] Heuck A., Schleiffer A., Clausen T. (2011). Augmenting β-augmentation: structural basis of how BamB binds BamA and may support folding of outer membrane proteins. J. Mol. Biol..

[60] Ruiz N., Falcone B., Kahne D., Silhavy T.J. (2005). Chemical conditionality: a genetic strategy to probe organelle assembly. Cell.

[61] Bryant J.A., Staunton K.A., Doherty H.M. (2023). BAM complex associated proteins in *Escherichia coli* are functionally linked to peptidoglycan biosynthesis, membrane fluidity and DNA replication. bioRxiv.

[62] Han L., Zheng J., Wang Y., Yang X., Liu Y., Sun C. (2016). Structure of the BAM complex and its implications for biogenesis of outer-membrane proteins. Nat. Struct. Mol. Biol..

[63] Bakelar J., Buchanan S.K., Noinaj N. (2016). The structure of the β-barrel assembly machinery complex. Science.

[64] Gentle I., Gabriel K., Beech P., Waller R., Lithgow T. (2004). The Omp85 family of proteins is essential for outer membrane biogenesis in mitochondria and bacteria. J. Cell Biol..

[65] Kozjak V., Wiedemann N., Milenkovic D., Lohaus C., Meyer H.E., Guiard B. (2003). An essential role of Sam50 in the protein sorting and assembly machinery of the mitochondrial outer membrane. J. Biol. Chem..

[66] Sommer M.S., Daum B., Gross L.E., Weis B.L., Mirus O., Abram L. (2011). Chloroplast Omp85 proteins change orientation during evolution. Proc. Natl. Acad. Sci. U. S. A..

[67] Misra R. (2012). Assembly of the β-barrel outer membrane proteins in Gram-negative bacteria, mitochondria, and chloroplasts. ISRN Mol. Biol..

[68] Lowry O.H., Rosebrough N.J., Farr A.L., Randall R.J. (1951). Protein measurement with the Folin phenol reagent. J. Biol. Chem..

[69] Leo J.C., Oberhettinger P., Linke D. (2015). Assessing the outer membrane insertion and folding of multimeric transmembrane β-barrel proteins. Methods Mol. Biol..

