# Supplementary material for: A minimum functional form of the Escherichia coli BAM complex constituted by BamADE assembles outer membrane proteins in vitro
Source: J Biol Chem. 2024 Apr 25;300(6):107324. doi: 10.1016/j.jbc.2024.107324 (PMC11130730; doi:10.1016/j.jbc.2024.107324)
Supplement: Supporting information [file mmc1.docx]

Supplementary materials

**Table S1.** The primers used in this study.

| Primer^1^ | Sequence (5’-3’) | Usage |
| --- | --- | --- |
| bamAs_s | TTCGCTACCGTAGCGCAGGCCGCTGAAGGGTTCGTAGTGAAAGA | For substrate protein BamA |
| bamAs _a | AGGATCCCCGGGTACCGAGCTCCCAGGTTTTACCGATGTTAAACT |  |
| bamA pTrc99a_s | TCTTTCACTACGAACCCTTCAGCGGCCTGCGCTACGGTAGCGAA |  |
| bamA pTrc99a_a | AGTTTAACATCGGTAAAACCTGGGAGCTCGGTACCCGGGGATCCT |  |
| ompA_s | AGGAAACAGACCATGGAATTCATGGTGAAAAAGACAGCTATCGCGA | For substrate protein OmpA |
| ompA_a | CAGGTCGACTCTAGAGGATCCTCAGTGGTGGTGGTGGTGGTGAGCCT  GCGGCTGAGTTACAACGTCT |  |
| bamAp_s | TATATCATATGGCGATGAAAAAGTTGC | For BamA over-expression |
| bamAp_a | TATATGCGGCCGCCCAGGTTTACCGATGTT |  |
| bamB _s | TATATCATATGCAATTGCGTAAATTACTGC | For BamB over-expression |
| bamB_ a | TATATGCGGCCGCACGTGTAATAGAGTACACGG |  |
| bamC _s | TATATCATATGGCTTACTCTGTTCAAAAGT | For BamC over-expression |
| bamC _a | TATATGCGGCCGCCTTGCTAAACGCAGCCT |  |
| bamD _s | TATATCATATGACGCGCATGAAATATCTGG | For BamD over-expression |
| bamD _a | TATATGCGGCCGCTGTATTGCTGCTGTTTGCG |  |
| bamE _s | TATATCATATGCGCTGTAAAACGCTG | For BamE over-expression |
| bamE _a | TATATGCGGCCGCGTTACCACTCAGCGCAGG |  |

^1^Forward and reverse primes are abbreviated as s and a, respectively.


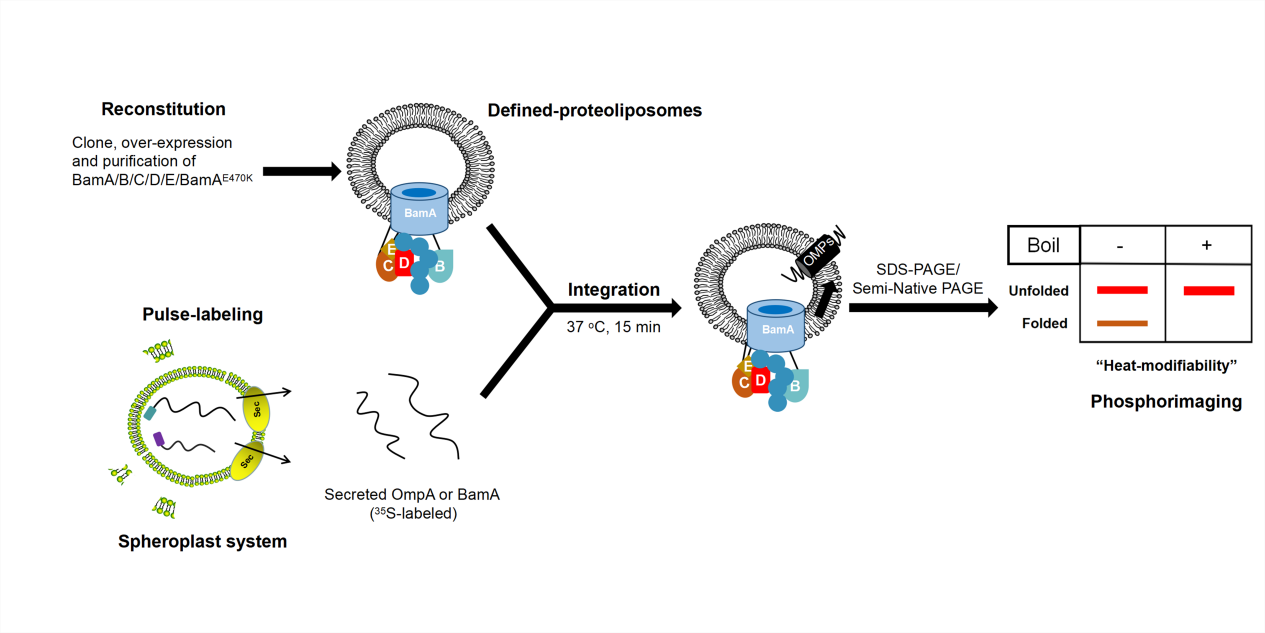


**Figure S1.** The experimental set-up used in the present study.

Based on a versatile system that we developed previously (1, 2), the experimental set-up used in the present work includes two parts: the well-defined proteoliposomes made from extensively purified BAM proteins that provide a clean outer membrane environment, and the spheroplast-secreted ^35^S-labeled model substrate OmpA/BamA protein that could *de novo* mimic the natural situation of OMPs before integration. By mixing the defined-proteoliposomes and the supernatant containing ^35^S-labeled OmpA/BamA after centrifugation of pulse-labeled spheroplasts, the reaction mixture was incubated at 37 ^o^C for 15 min to enable the occurance of integration. The sample was then analyzed by SDS-PAGE (OmpA) or Semi-Native PAGE (BamA) and visualized with a GE Typhoon Imager. The cartoon gel refers to the heat-modifiability of β-barrel proteins, which run faster when treated at low temperatures representing the successful integration into the membranes (marked with “Folded”), while when boiled in the presence of SDS, the folded OmpA (or BamA) becomes completely denatured and thus run slower (marked with “Unfolded”).

**Reference**

1. Norell, D., Heuck, A., Tran-Thi, T. A., Götzke, H., Jacob-Dubuisson, F., Clausen, T. et al. (2014) Versatile in vitro system to study translocation and functional integration of bacterial outer membrane proteins. *Nat Commun*. **5**, 5396

2. Fan, E., Norell, D., and Müller, M. (2015) An *in vitro* assay for substrate translocation by FhaC in liposomes. *Methods Mol Biol.* **1329**, 111-125
